# Draft genome sequence of *emm*103/ST1363 *Streptococcus pyogenes* strain AB1, isolated from the blood of a woman with peritonitis and toxic shock syndrome

**DOI:** 10.1128/mra.01027-23

**Published:** 2024-01-30

**Authors:** Takahiro Maeda, Haruno Yoshida, Noriyuki Abe, Koichiro Murakami, Mieko Goto, Takashi Takahashi

**Affiliations:** 1 Laboratory of Infectious Diseases, Graduate School of Infection Control Sciences, Ōmura Satoshi Memorial Institute, Kitasato University, Minato, Tokyo, Japan; 2 Department of Clinical Laboratory Medicine, Tenri Hospital, Tenri, Nara, Japan; 3 Department of General Internal Medicine, Tenri Hospital, Tenri, Nara, Japan; Wellesley College Department of Biological Sciences, USA

**Keywords:** *Streptococcus pyogenes*, woman, peritonitis, toxic shock syndrome

## Abstract

We report the draft genome sequence of *Streptococcus pyogenes* strain AB1 isolated from the blood of a woman with peritonitis—toxic shock syndrome. The genome measured 1.855 Mbp, with a G + C content of 38.3%. Sequences unmapped to the reference genome sequence of M1 SF370 (GenBank accession number AE004092.2) were characterized.

## ANNOUNCEMENT

*Streptococcus pyogenes* strain AB1 harboring *emm*103/ST1363 was isolated from the standard blood cultures of a 22-year-old woman presenting with primary peritonitis, bilateral empyema, and toxic shock syndrome. The research is carried out in accordance with the Declaration of Helsinki. Informed consent was obtained from the patient and her family for this case presentation. We report the draft genome sequence of AB1, with characteristic sequences unmapped to the reference genome sequence of SF370.

The strain AB1 was inoculated in 5% sheep blood agar plate and aerobically incubated in 5% CO_2_ at 35°C for 24 h. A colony with a gray-white smooth appearance, indicating β-hemolysis, was picked up from the plate and grown overnight in Todd–Hewitt broth supplemented with yeast extract. DNA was extracted using a DNeasy Blood & Tissue Kit (Qiagen, Germany) after pretreatment with proteinase K (1–3). Whole-genome sequencing was performed on a DNBSEQ-G400RS platform (MGI-Tech, Japan) using DNA Nanoball technology based on circular DNA fragment amplification (4). The sequencing library was generated using MGIEasy FS DNA Library Prep Set (item no. 1000006987, MGI-Tech). Paired-end runs were performed with a read length of 2 × 150 bp.

Sequencing yielded 19,820,691 reads (5.946 Gbp). The reads were trimmed using the quality trimming tool in CLC Genomics Workbench (ver. 23.0.4) with default parameters. *De novo* assembly was performed using the CLC Genomics Workbench with modified parameters, wherein the minimum contig length was set to 800 bp. Draft genome sequences were annotated using the DDBJ Fast Annotation and Submission Tool (DFAST; https://dfast.nig.ac.jp) (5). The assembly metrics and annotated features included genome size (1,855,299 bp), number of contigs (56), average coverage (3,205×), *N*_50_ (149,366 bp), number of coding DNA sequences (CDSs)/tRNAs/rRNAs/clustered regularly interspaced short palindromic repeats (1,758/27/5/1), G + C content (38.3%), and coding ratio (84.8%).

Mapping of AB1 reads to the reference genome sequence (GenBank accession number AE004092.2) of serotype M1 *S. pyogenes* strain SF370 was performed using the reference tool with default parameters in the CLC Genomics Workbench. The remaining unmapped reads (2,911,962 reads) were assembled *de novo*, which yielded 47 contigs. These contigs were uploaded to the web-based applications PathogenFinder ver. 1.1 (https://cge.food.dtu.dk/services/PathogenFinder/) (6) and DFAST to identify pathogenic gene families that were not present in SF370. In all, 16 CDSs (length 1,206–144 bp) were recognized by both applications (Table 1). These CDSs encoded a transcriptional regulator (contig number 12) (7), DNA integrase (contig number 1), and phage/phage-associated proteins (main contig number 4) (8, 9), identical to those of *S. pyogenes* and *S. dysgalactiae* subsp. *equisimilis* (SDSE). CDS-encoding DNA integrase from SDSE has been observed previously in the genome sequence of blood-origin *S. canis* isolated from a dog with necrotizing soft tissue infection (3), suggesting genetic transmission between *S. pyogenes* and other pathogenic streptococci.

**TABLE 1:** Traits of *Streptococcus pyogenes* strain AB1^*a*^ pathogenic gene families that were not present in serotype M1 SF370,^*b*^ identical to pathogenic streptococcus

Data from PathogenFinder ver. 1.1^*c*^								Data from DFAST^*e*^ and BLASTp^*f*^	
	Pathogenic streptococcus ^*d*^	Accession no. (Protein no.)	Product	Contig no.	Nucleotide start	Nucleotide end	Size (bp)	Product	Product
1	*S. pyogenes* MGAS9429	CP000259.1 (ABF31304.1)	Transcriptional regulator, AraC family	12	38,071 (reverse)	36,866	1,206	YSIRK-targeted surface antigen transcriptional regulator	Bacterial regulatory helix-turn-helix, AraC family protein
2 2	*S. dysgalactiae* subsp. *equisimilis* GGS_124	AP010935.1 (BAH82621.1)	DNA integration/recombination/inversion protein	1	35,315	36,478	1,164	Site-specific integrase	Site-specific integrase
3	*S. pyogenes* MGAS9429	CP000259.1 (ABF31725.1)	Phage protein	4	315,992	316,633	642	Hypothetical protein	Hypothetical protein
4	*S. pyogenes* MGAS6180	CP000056.2 (AAX72126.1)	Phage protein	4	311,016 (reverse)	310,534	483	Hypothetical protein	DUF669 domain-containing protein in phage proteins
5	*S. pyogenes* MGAS10394	CP000003.1 (AAT87334.1)	Holin	4	280,566 (reverse)	280,111	456	Hypothetical protein	Phage holin family protein
6	*S. pyogenes* MGAS9429	CP000259.1 (ABF31767.1)	Phage protein	4	293,210 (reverse)	292,785	426	Hypothetical protein	Hypothetical protein
7	*S. pyogenes* MGAS6180	CP000056.2 (AAX72112.1)	Phage protein	4	301,081	301,458	378	Type II toxin-antitoxin system HicB family antitoxin	Type II toxin-antitoxin system HicB family antitoxin
8	*S. pyogenes* MGAS6180	CP000056.2 (AAX72904.1)	Phage protein	27	22	429	408	Hypothetical protein	Hypothetical protein
9	*S. pyogenes* MGAS8232	AE009949.1 (AAL97901.1)	Hypothetical phage protein	4	313,944 (reverse)	313,615	330	Hypothetical protein	Hypothetical protein
10	*S. pyogenes* MGAS10394	CP000003.1 (AAT87160.1)	Unknown phage protein	4	318,467	318,772	306	Hypothetical protein	Membrane protein
11	*S. pyogenes* MGAS8232	AE009949.1 (AAL97899.1)	Hypothetical phage protein	4	313,424 (reverse)	313,140	285	Hypothetical protein	Hypothetical protein
12	*S. pyogenes* MGAS10394	CP000003.1 (AAT87144.1)	Unknown phage protein	4	297,702 (reverse)	297,478	225	Hypothetical protein	Hypothetical protein
13	*S. pyogenes* NZ131	CP000829.1 (ACI60688.1)	Hypothetical protein	1	26,121 (reverse)	25,978	144	Hypothetical protein	Hypothetical protein
14	*S. pyogenes* MGAS10270	CP000260.1 (ABF33634.1)	Phage protein	4	300,844	301,029	186	Hypothetical protein	Toxin-antitoxin system, toxin component, HicA family
15	*S. pyogenes* MGAS10394	CP000003.1 (AAT87133.1)	Unknown phage protein	4	291,758 (reverse)	291,585	174	Hypothetical protein	Hypothetical protein
16	*S. pyogenes* MGAS10394	CP000003.1 (AAT87145.1)	Unknown phage protein	4	297,865 (reverse)	297,695	171	Hypothetical protein	Hypothetical protein

^*a*^ GenBank nucleotide accession number BTGW00000000.1. GenBank assembly accession number GCF_033008475.1.

^*b*^ GenBank nucleotide accession number AE004092.2. GenBank assembly accession number GCF_000006785.2.

^*c*^ The PathogenFinder ver. 1.1 (https://cge.cbs.dtu.dk/services/PathogenFinder/, Center for Genomic Epidemiology) was applied to obtain an overview of the genomic pathogenic gene families.

^*d*^ We found the data regarding the pathogenic streptococcus, GenBank accession number, and protein number using the PathogenFinder ver. 1.1.

^*e*^ The DDBJ Fast Annotation and Submission Tool (DFAST; https://dfast.nig.ac.jp/) was used to confirm the product (coding DNA sequence) obtained using PathogenFinder ver. 1.1.

^*f*^ The Protein Basic Local Alignment Search Tool (BLASTp; https://blast.ncbi.nlm.nih.gov/Blast.cgi) of the National Center for Biotechnology Information was also applied to confirm the product (coding DNA sequence) obtained using PathogenFinder ver. 1.1.

We determined the phage/phage-associated protein distribution and antimicrobial resistance (AMR) genotypes by the phage detection application PHASTER (https://phaster.ca) (10) and ResFinder ver. 4.1 (https://cge.food.dtu.dk/services/ResFinder/) (11) using whole-contigs. AB1 contained a phage-related region (51.6 kbp) of contig 4 without AMR genes.

## Data Availability

The draft genome sequence is deposited in DDBJ/EMBL/GenBank under the accession number BTGW00000000.1, with the SRA accession number DRR493916.
